# Supplementary material for: Raman Enhancement of Nanoparticle Dimers Self-Assembled Using DNA Origami Nanotriangles
Source: Molecules. 2021 Mar 17;26(6):1684. doi: 10.3390/molecules26061684 (PMC8002687; doi:10.3390/molecules26061684)

Raman Enhancement of Nanoparticle Dimers Self-Assembled using DNA Origami Nanotriangles

Sergio Kogikoski Jr.[1,2], Kosti Tapio[1], Robert Edler von Zander[1], Peter Saalfrank[1], Ilko Bald[1*]

[1] Institute of Chemistry, University of Potsdam, Potsdam, Germany;

[2] Department of Analytical Chemistry, Institute of Chemistry, State University of Campinas – UNICAMP, PO Box 6154, 13084-974, Campinas, SP, Brazil;

*e-mail: bald@uni-potsdam.de

Tel: +49 331-977-5238 (IB)

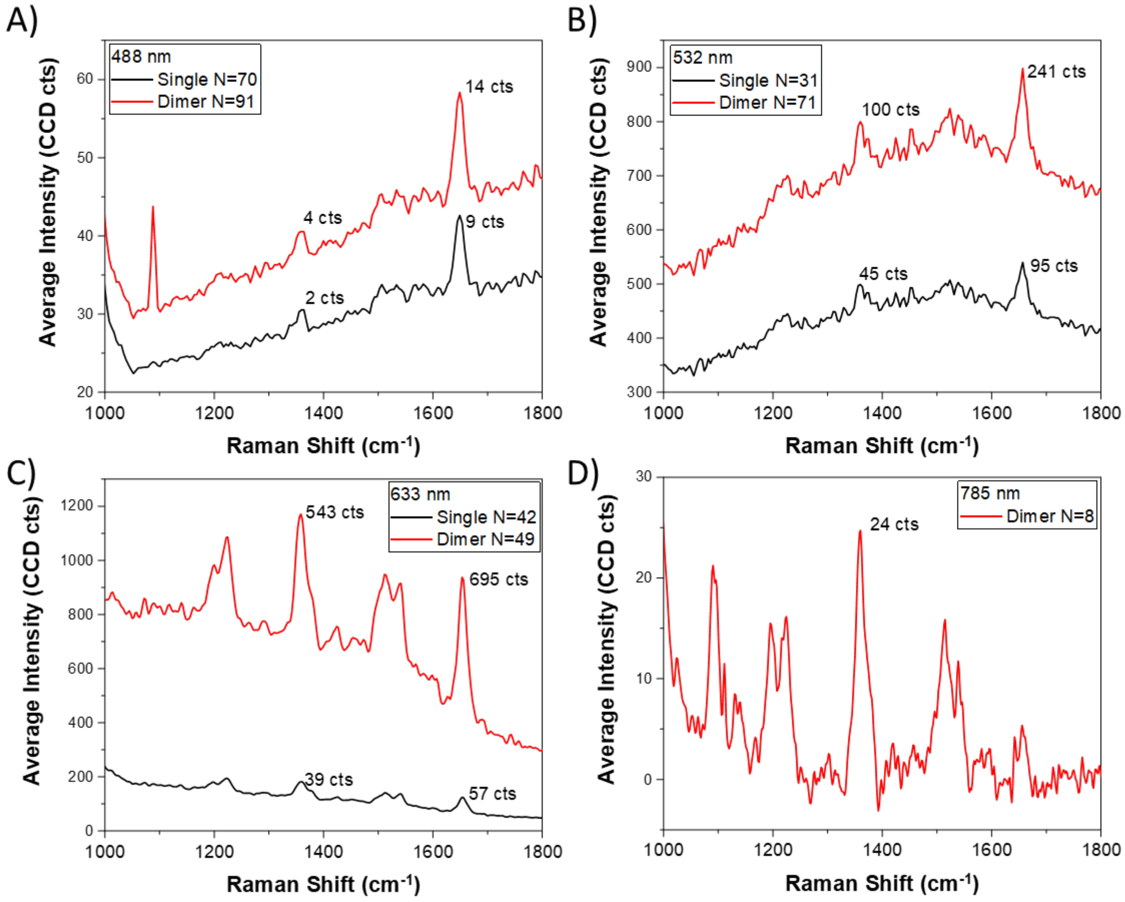

Figure S1. Averaged SERS spectra of self-assembled dimers or single nanoparticles composed of 80 nm AuNP modified with TAMRA. On the graphs inset the number of spectra that were obtained and averaged is given. The exciting wavelengths used were A) 488 nm, B) 532 nm, C) 633 nm and D) 785 nm. In all Raman experiments the laser power was kept at 300 µW and the integration time was 2 s.

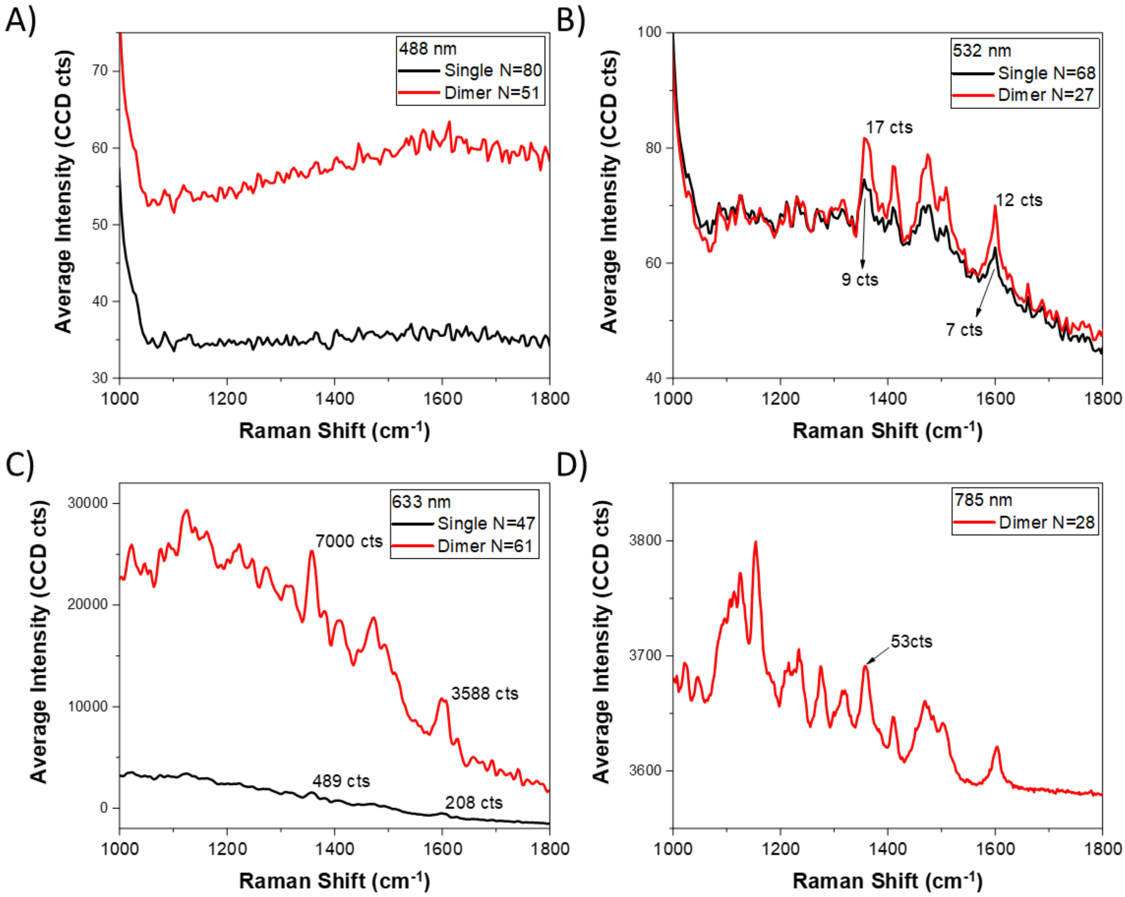

Figure S2. Averaged SERS spectra of self-assembled dimers or single nanoparticles composed of 80 nm AuNP modified with Cy5. On the graphs inset the number of spectra that were obtained and averaged is given. The exciting wavelengths used were A) 488 nm, B) 532 nm, C) 633 nm and D) 785 nm. In all Raman experiments the laser power was kept at 300 µW and the integration time was 2 s.

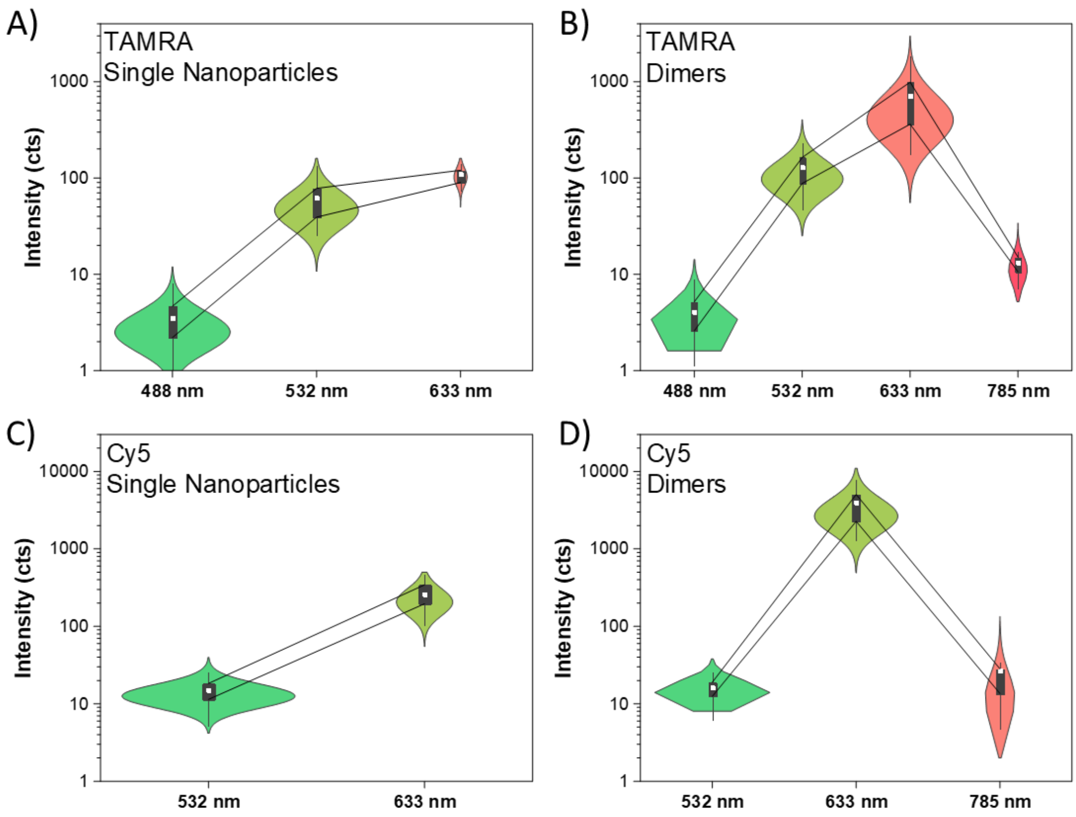

Figure S3. A) and B) Intensity distribution for the 1360 cm^{-1} peak region of all obtained spectra at the excitation wavelengths used for the AuNP 60 nm single nanoparticle and for dimers, respectively, modified with TAMRA dyes. C) and D) Intensity distribution for the 1380 cm^{-1} peak region of all obtained spectra at the excitation wavelengths used for the single nanoparticle and for dimers, respectively, modified with Cy5 dyes.

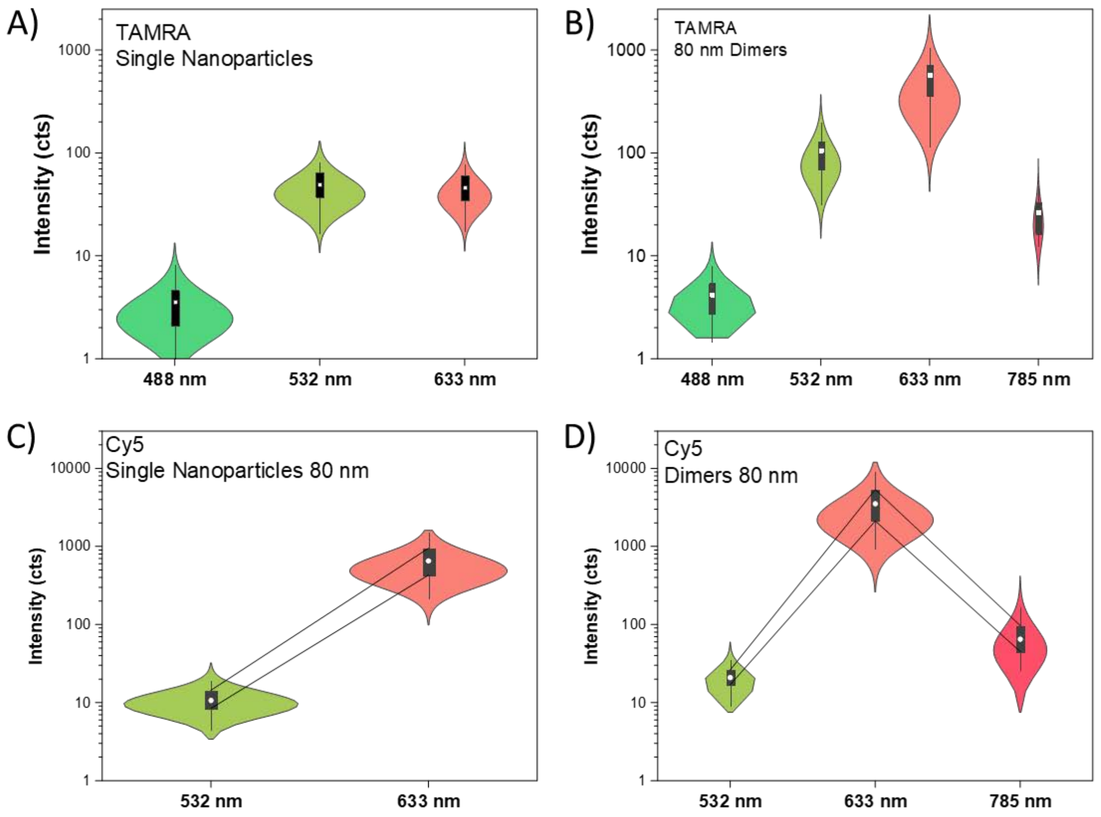

Figure S4. A) and B) Intensity distribution for the 1360 cm^{-1} peak region of all obtained spectra at the excitation wavelengths used for the AuNP 80 nm single nanoparticle and for dimers, respectively, modified with TAMRA dyes. C) and D) Intensity distribution for the 1380 cm^{-1} peak region of all obtained spectra at the excitation wavelengths used for the single nanoparticle and for dimers, respectively, modified with Cy5 dyes.

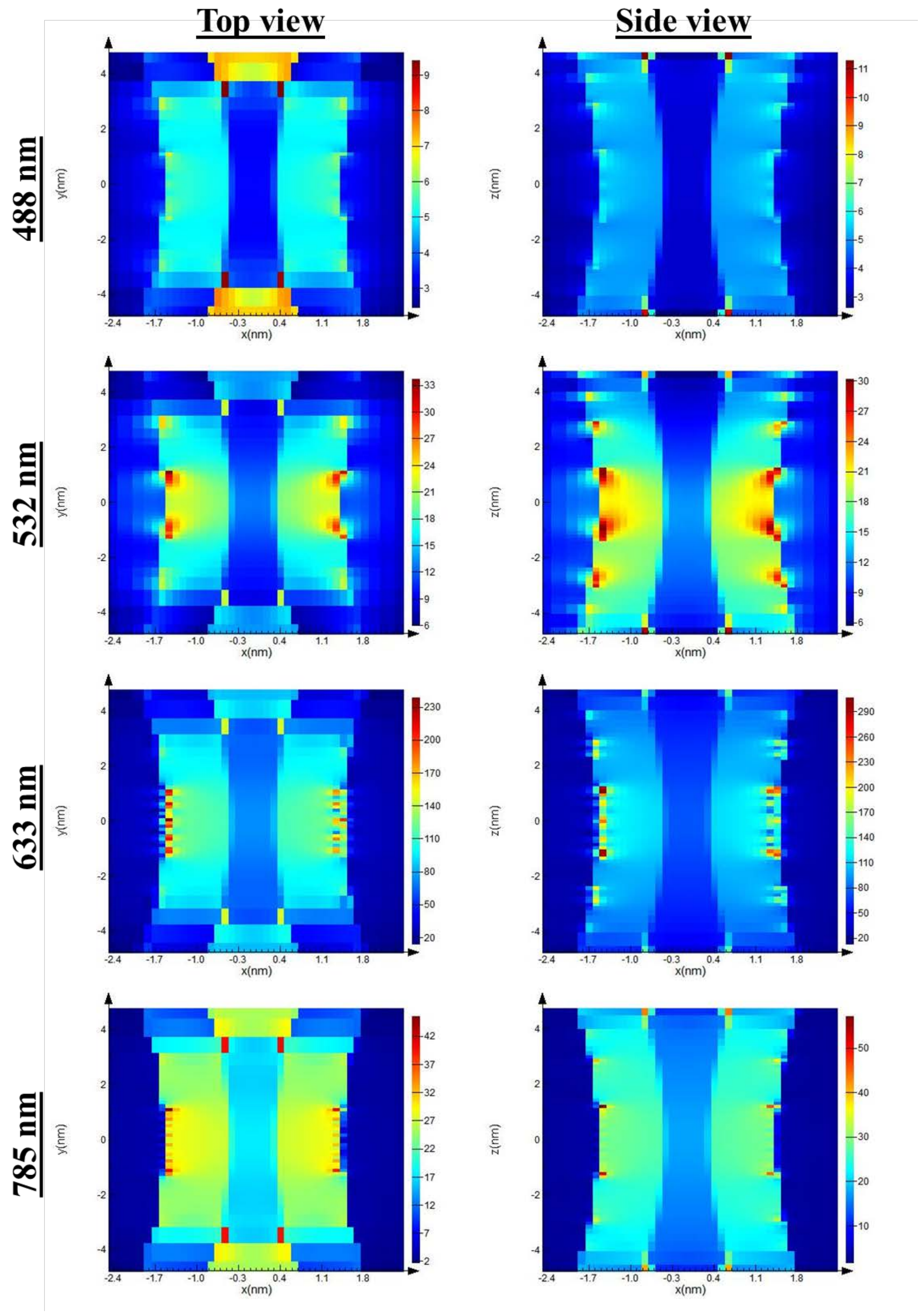

Figure S5. A) and B) FDTD simulated enhancement field map in the gap of a dimer composed by DNA coated 60 nm AuNP on a silicon substrate in both top and side view. The gap distance is constant at 3.5 nm between NP surfaces, and the excitation wavelength used is given for each data pair. The polarization of the incident light is along the axis of the gap.

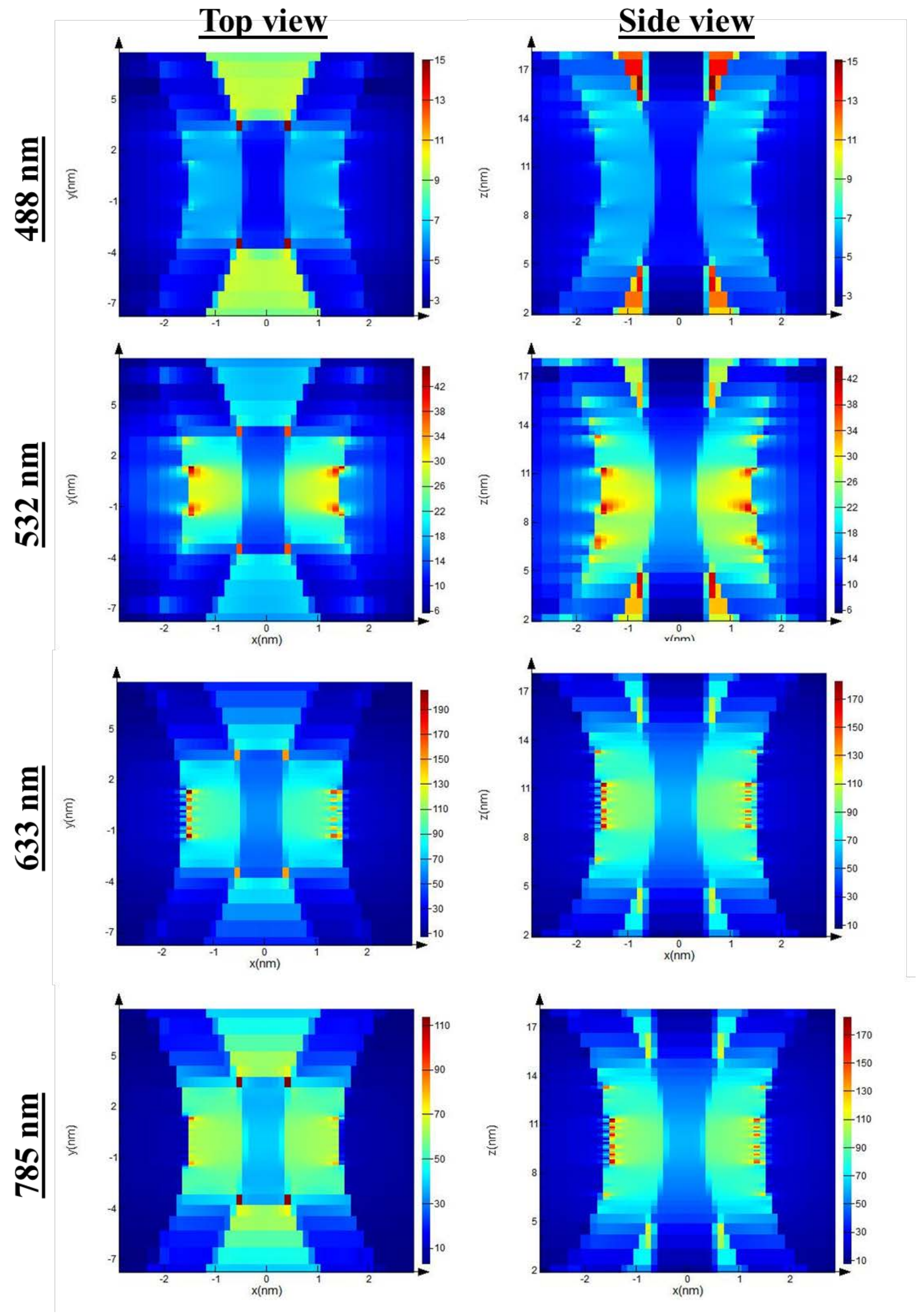

Figure S6. A) and B) FDTD simulated enhancement field map in the gap of a dimer composed by DNA coated 80 nm AuNP on a silicon substrate in both top and side view. The gap distance is constant at 3.5 nm between NP surfaces, and the excitation wavelength used is given for each data pair. The polarization of the incident light is along the axis of the gap.

SEM+SERS CORRELATION DATA

On the figures it is given the correlation between the SEM and SERS maps, the circled marks are referred to single nanoparticle and the dots to the correlated dimers spectra. All the extracted data from the maps are given in the plots. The comparison of the averaged spectra of the single particles and dimers is also given.

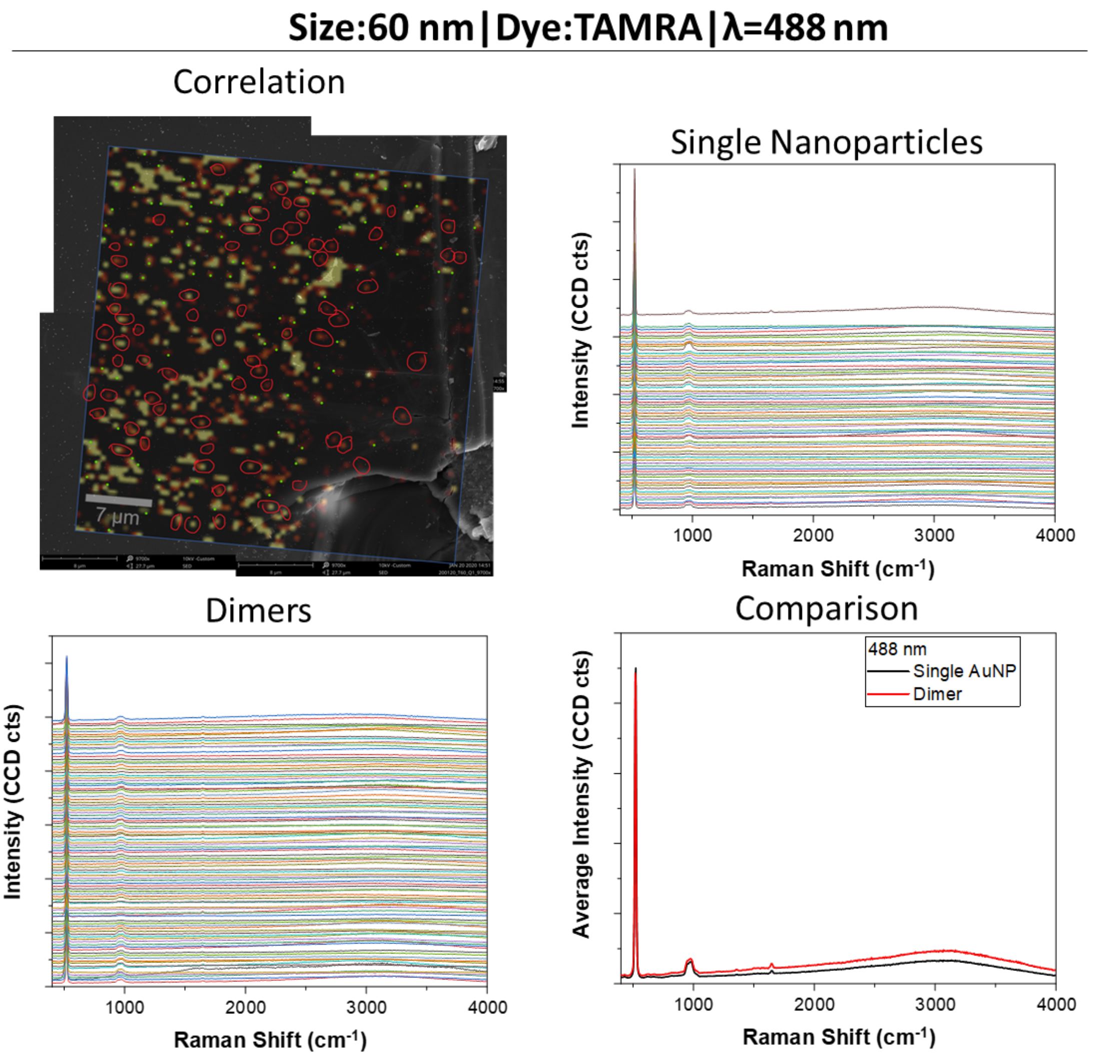

Size:60 nm|Dye:TAMRA|λ=532 nm

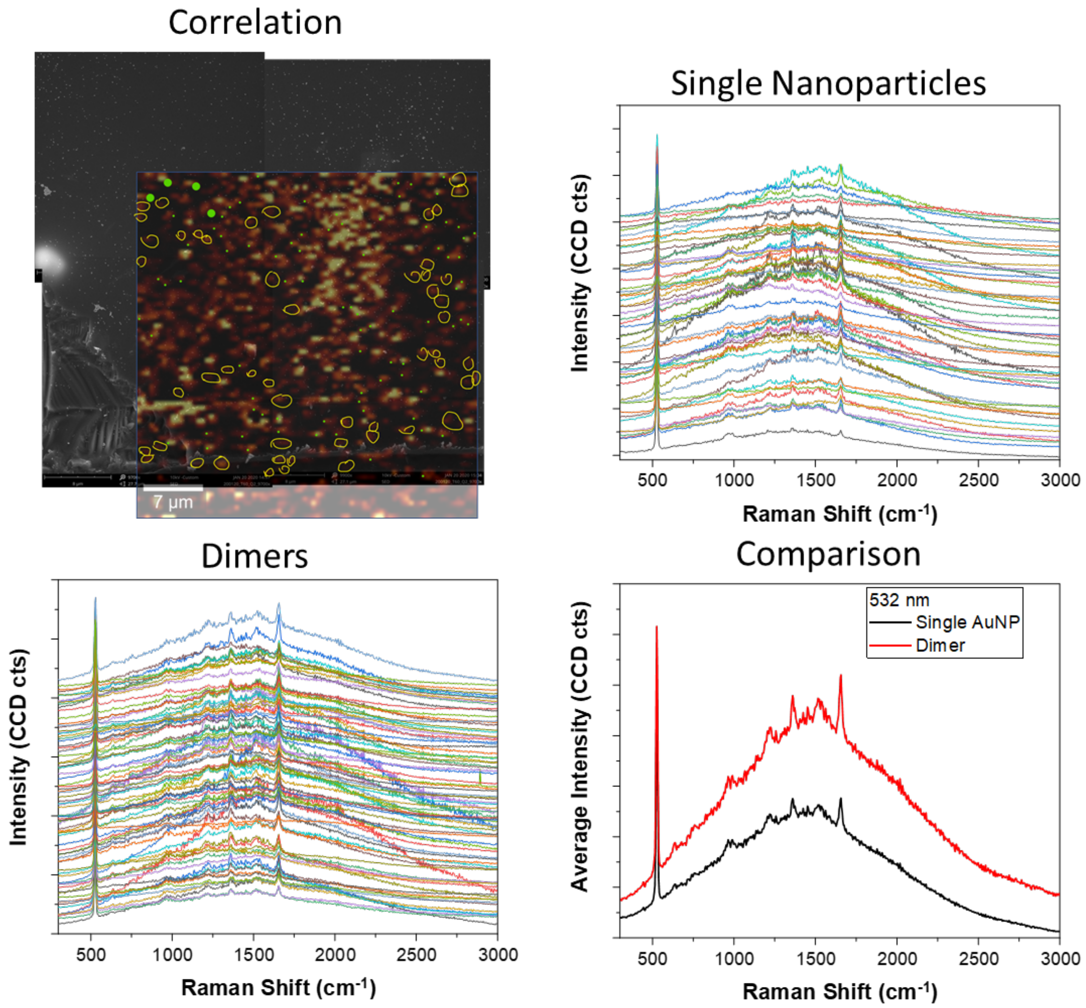

Size:60 nm|Dye:TAMRA|λ=633 nm

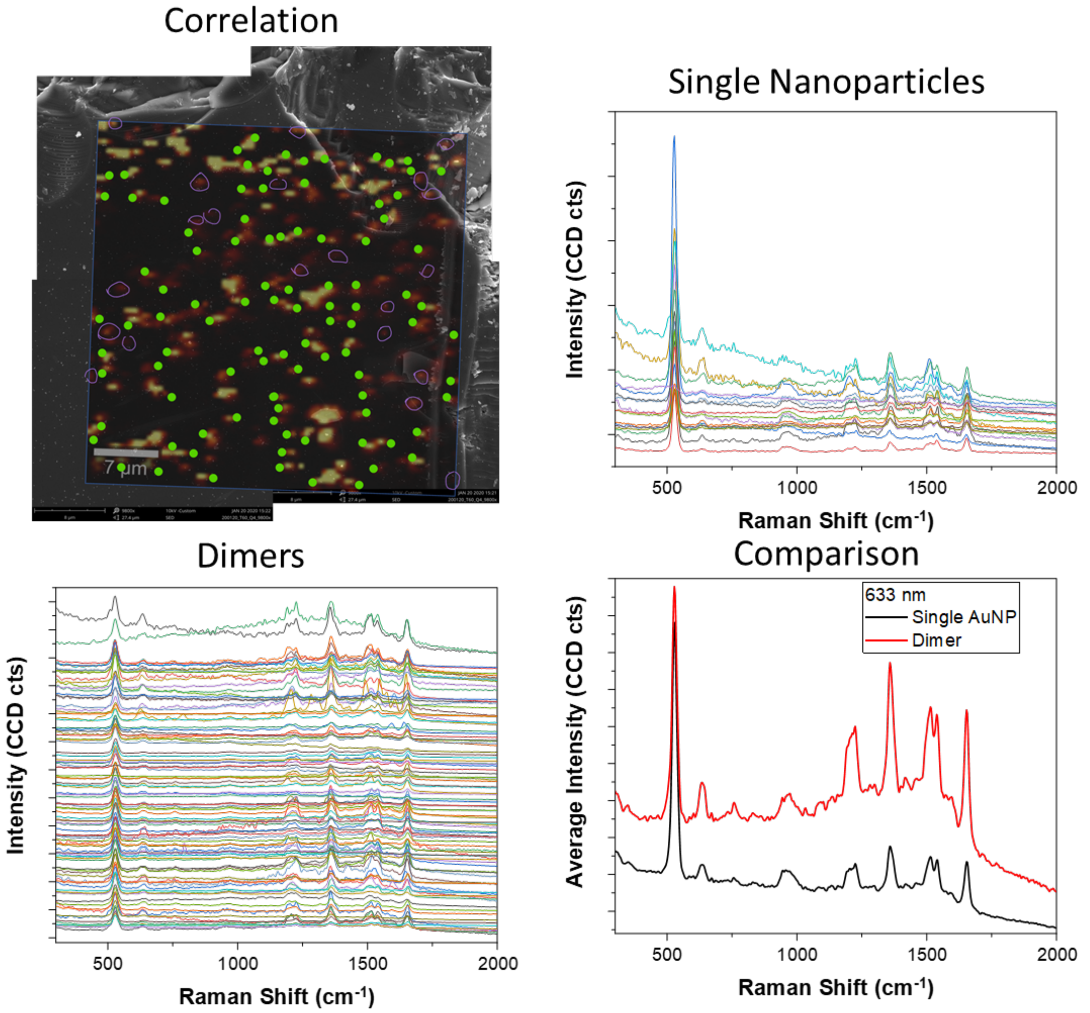

Size:60 nm|Dye:TAMRA|λ=785 nm

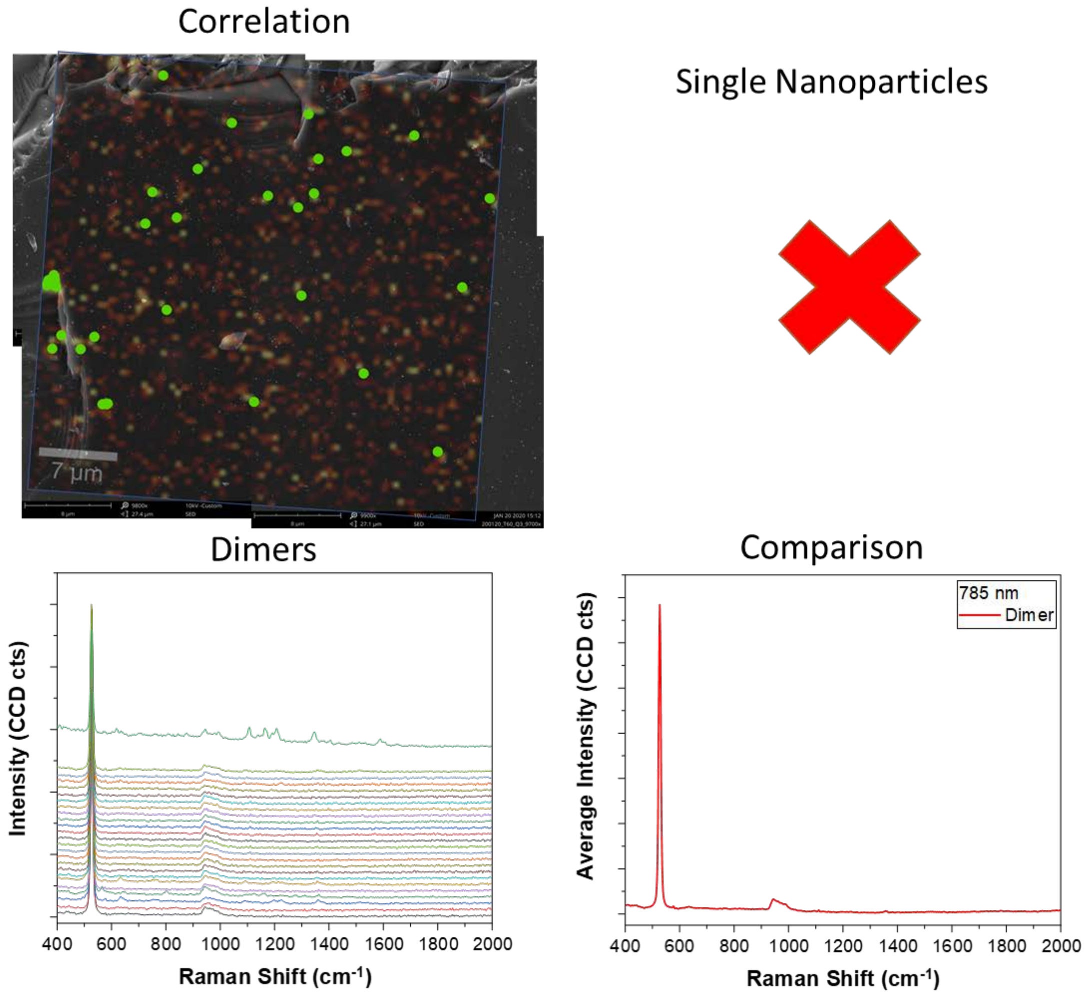

Size:80 nm|Dye:TAMRA|λ=488 nm

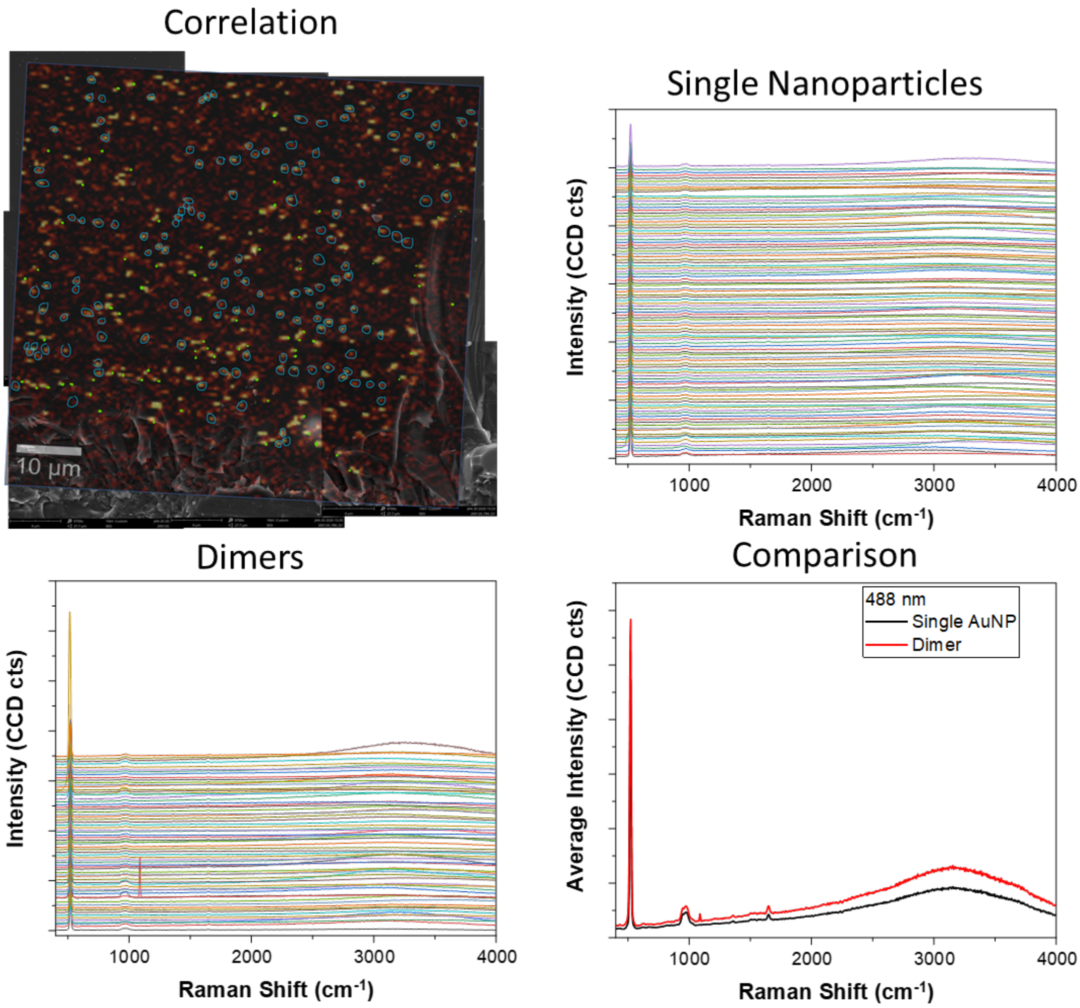

Size:80 nm|Dye:TAMRA|λ=532 nm

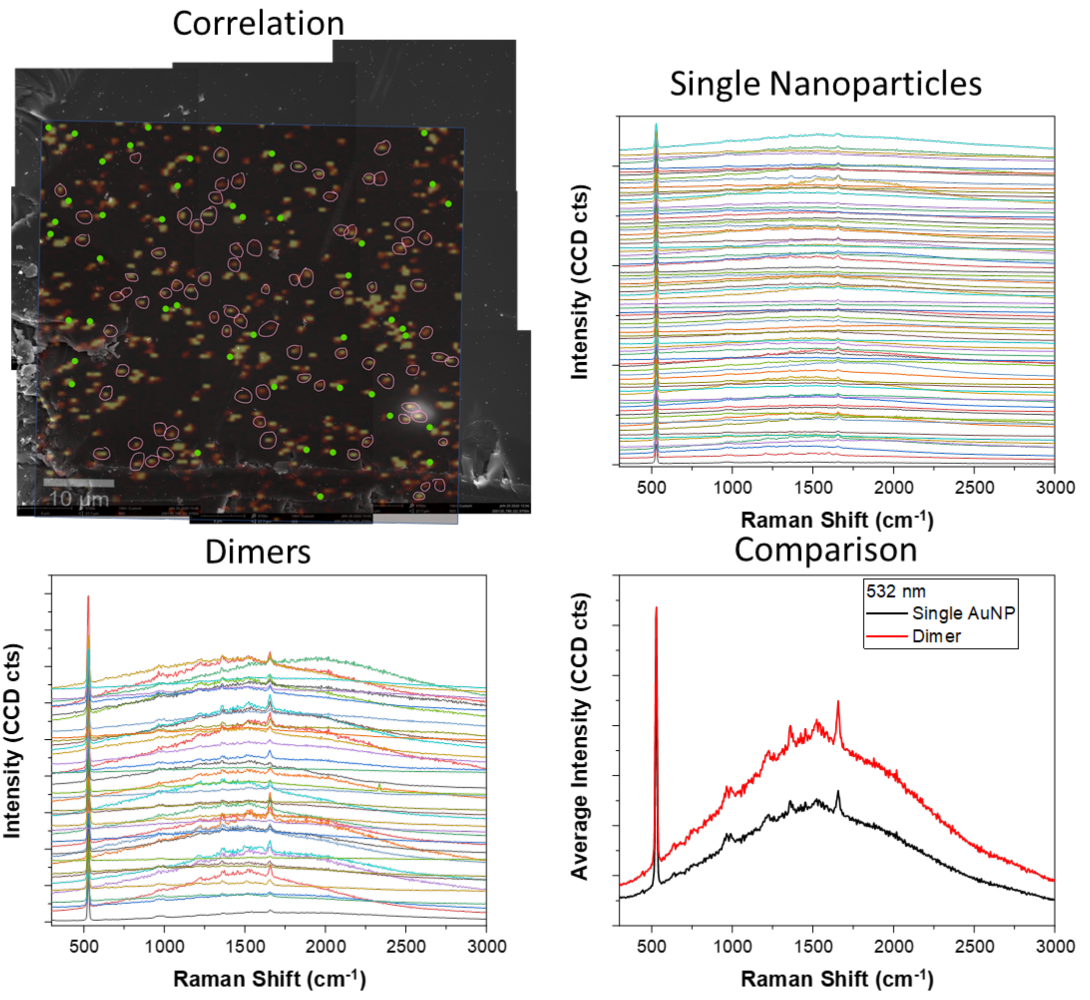

Size:80 nm|Dye:TAMRA|λ=633 nm

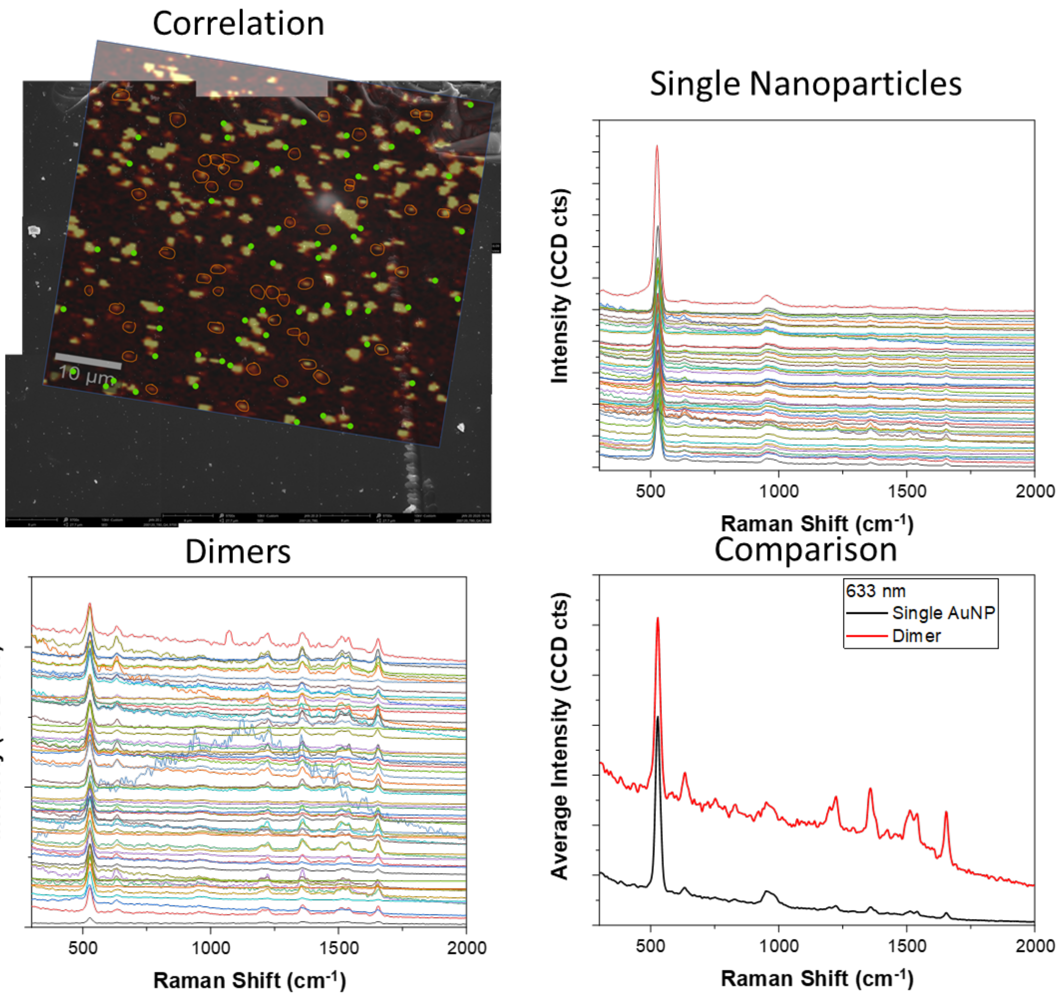

Size:80 nm|Dye:TAMRA|λ=785 nm

Correlation

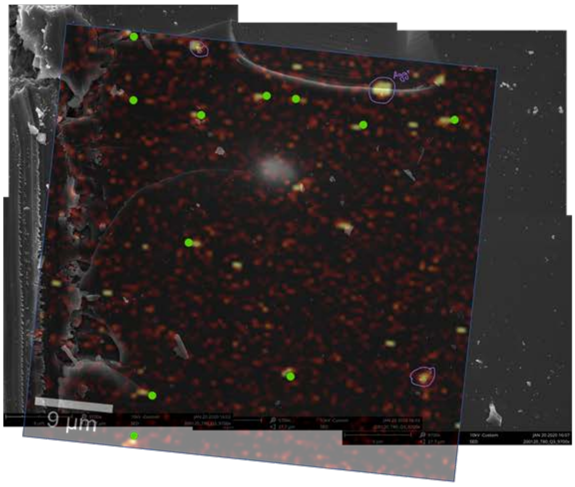

Single Nanoparticles

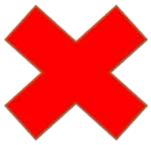

Dimers

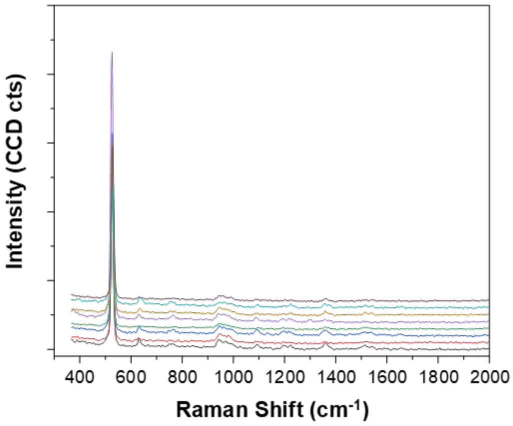

Comparison

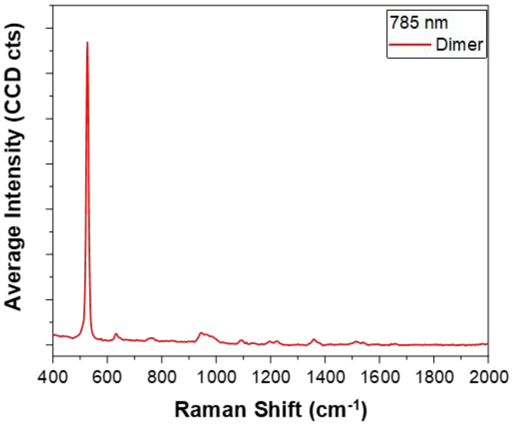

Size:60 nm|Dye:Cy5|λ=488 nm

Correlation

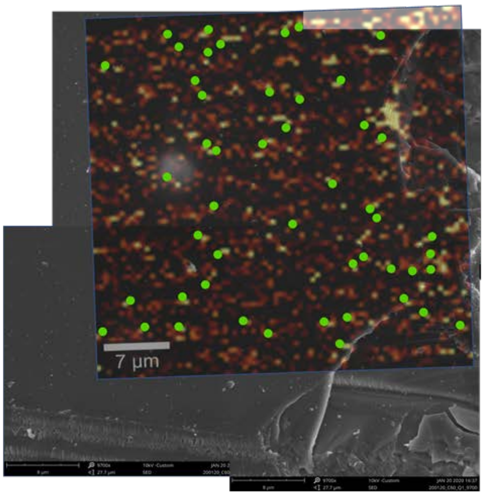

Single Nanoparticles

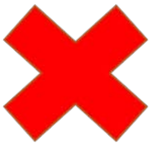

Dimers

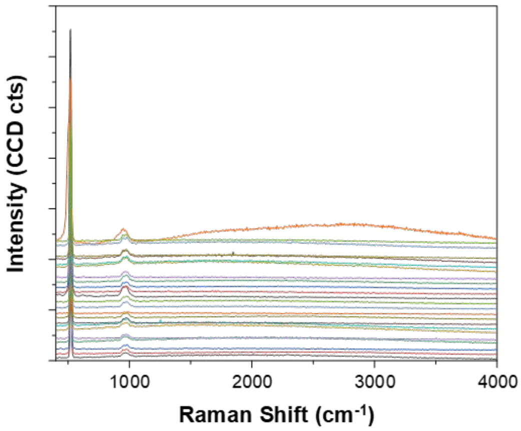

Comparison

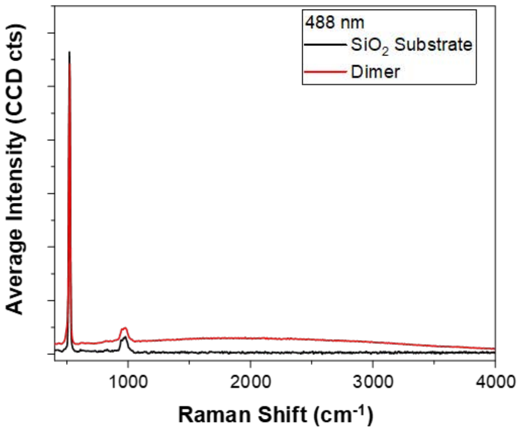

Size:60 nm|Dye:Cy5|λ=532 nm

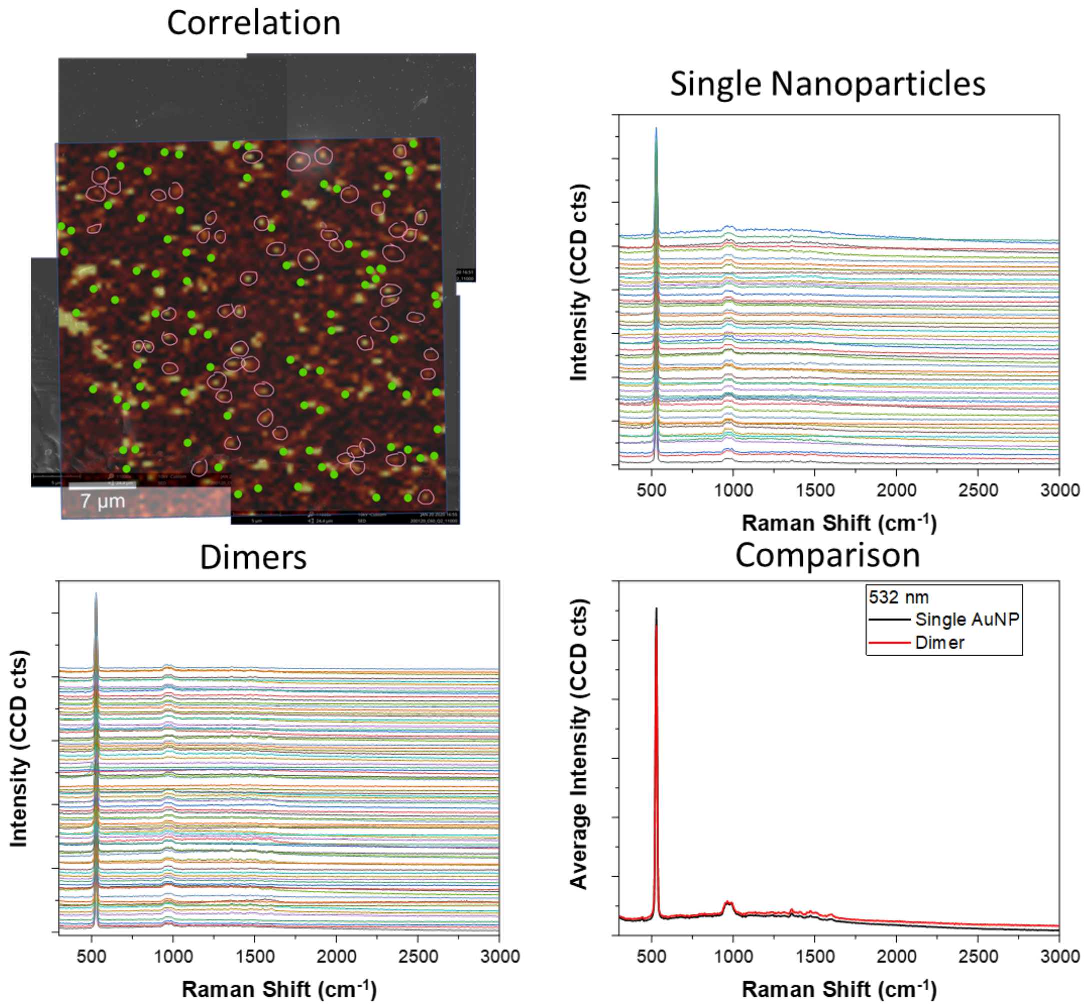

Size:60 nm|Dye:Cy5|λ=633 nm

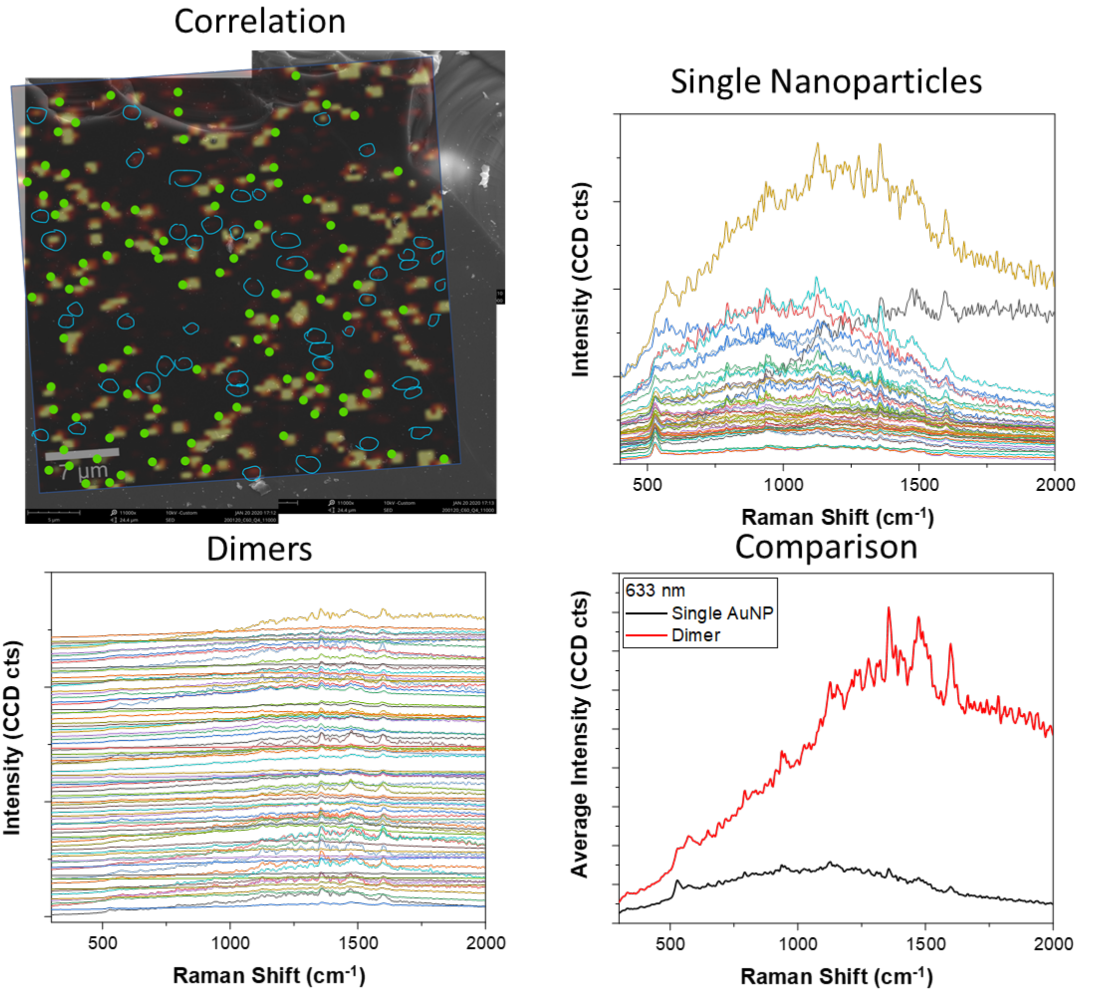

Size:60 nm|Dye:Cy5|λ=785 nm

Correlation

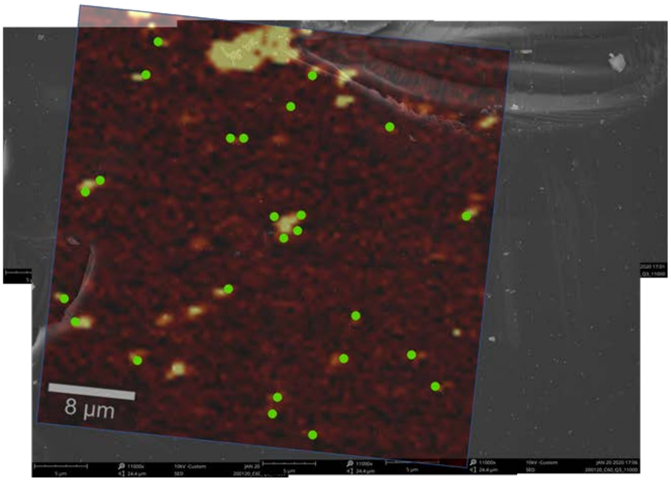

Single Nanoparticles

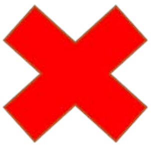

Dimers

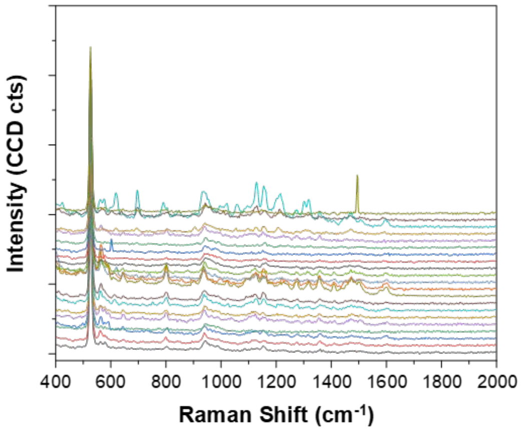

Comparison

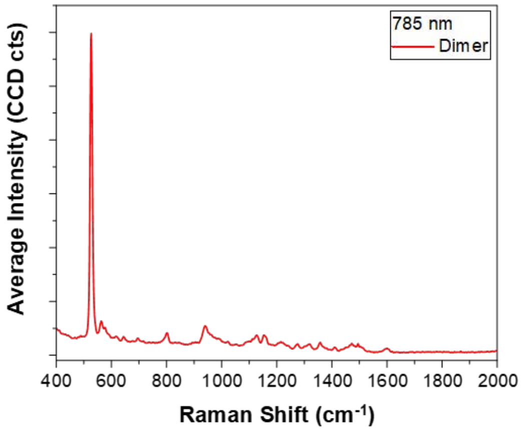

Size:80 nm|Dye:Cy5|λ=488 nm

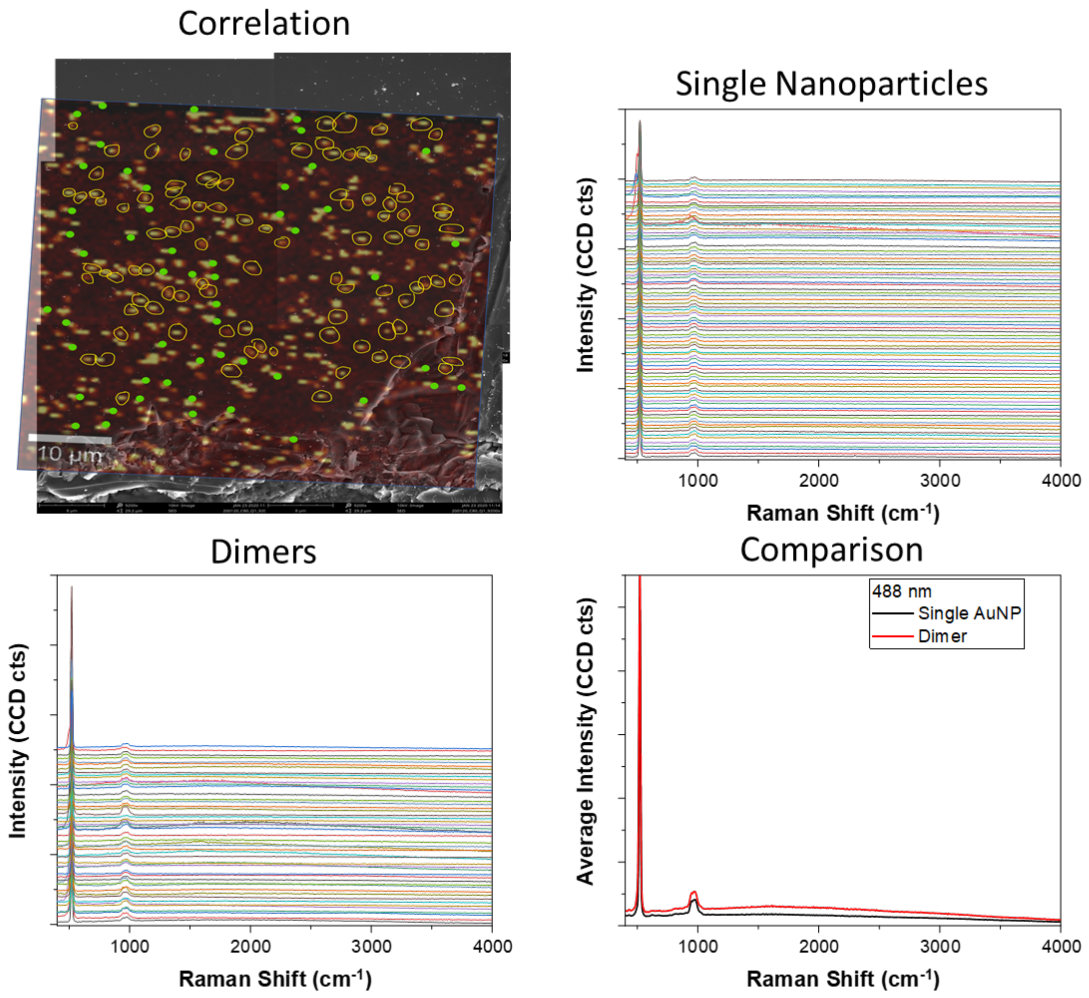

Size:80 nm|Dye:Cy5|λ=532 nm

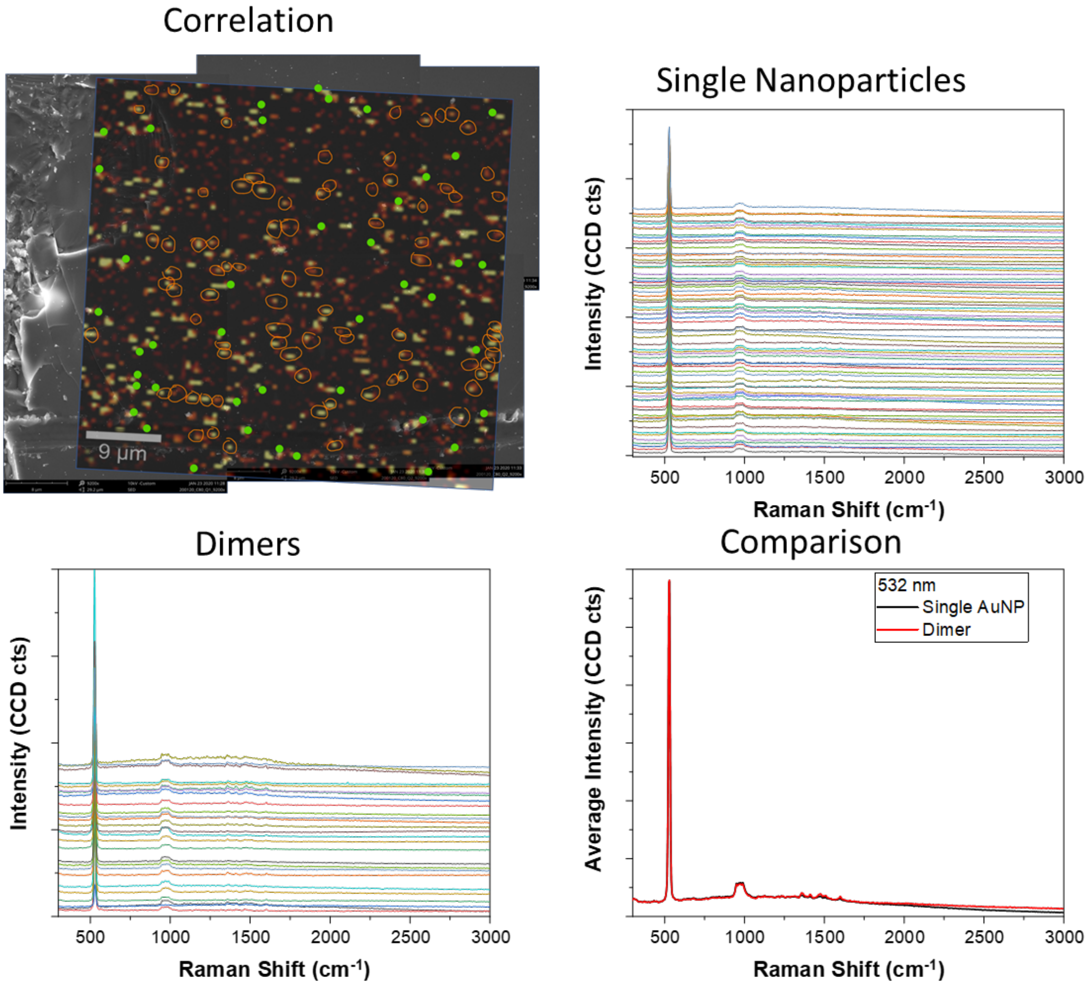

Size:80 nm|Dye:Cy5|λ=633 nm

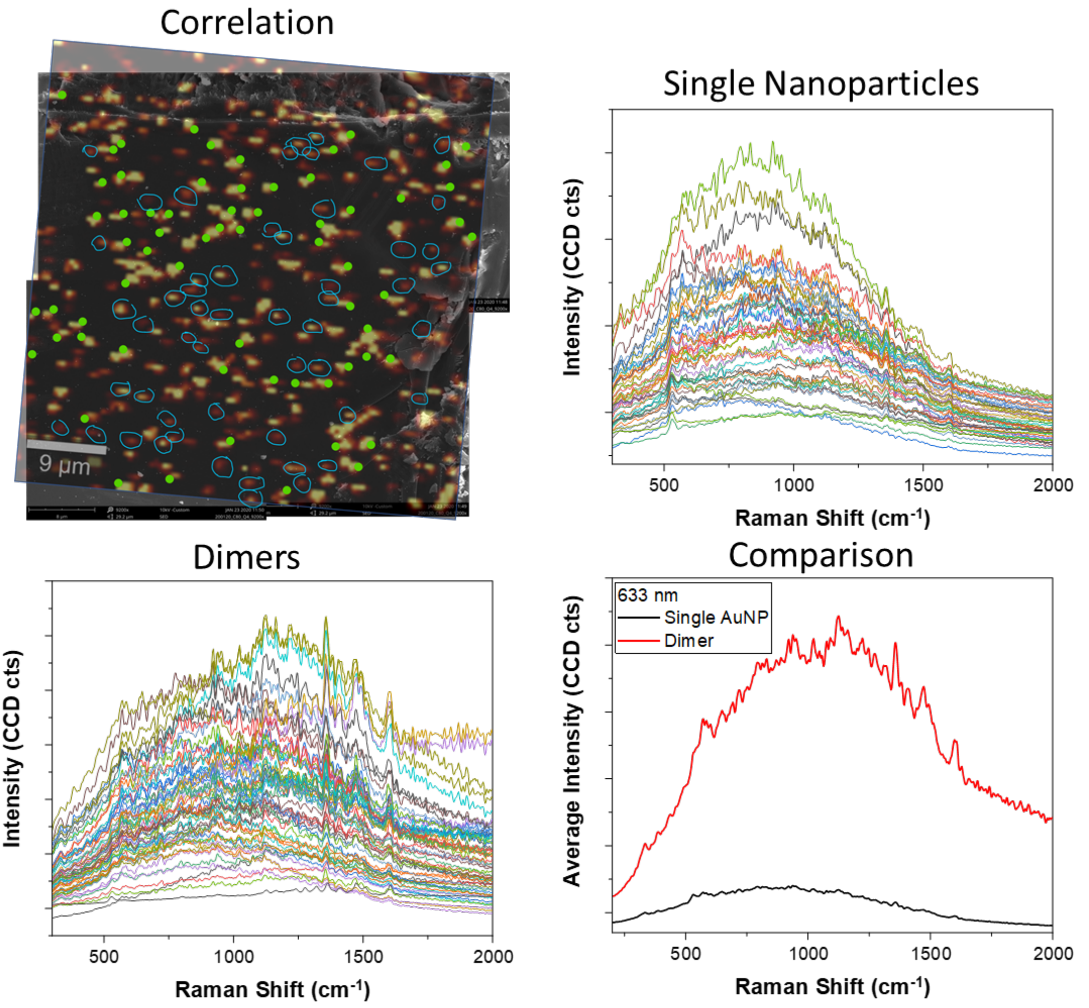

Size:80 nm|Dye:Cy5|λ=785 nm

Correlation

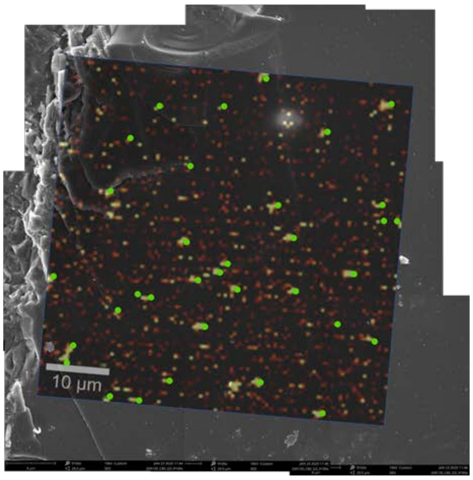

Single Nanoparticles

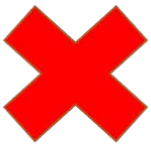

Dimers

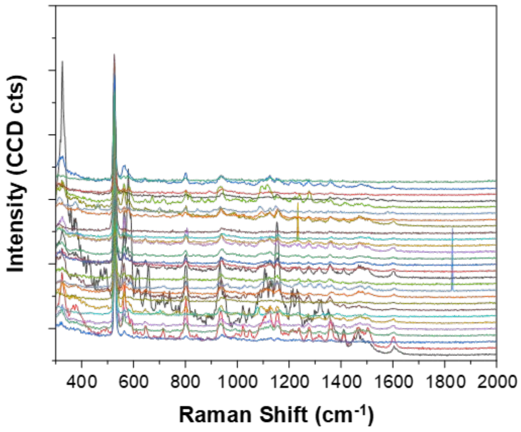

Comparison

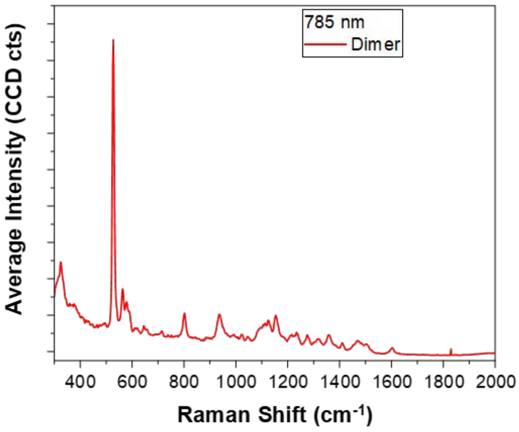

Supplement: Supplementary file 1 [file molecules-26-01684-s001.pdf]
